# The Protozoan Inhibitor Atovaquone Affects Mitochondrial Respiration and Shows *In Vitro* Efficacy Against Glucocorticoid-Resistant Cells in Childhood B-Cell Acute Lymphoblastic Leukaemia

**DOI:** 10.3389/fonc.2021.632181

**Published:** 2021-03-15

**Authors:** Yordan Sbirkov, Tsvetomira Ivanova, Hasan Burnusuzov, Kalina Gercheva, Kevin Petrie, Tino Schenk, Victoria Sarafian

**Affiliations:** ^1^ Department of Medical Biology, Medical University of Plovdiv, Plovdiv, Bulgaria; ^2^ Research Institute at Medical University of Plovdiv, Medical University of Plovdiv, Plovdiv, Bulgaria; ^3^ Department of Pediatrics and Medical Genetics, Medical University of Plovdiv, Plovdiv, Bulgaria; ^4^ Center for Competence Personalized Innovative Medicine (PERIMED), Medical University of Plovdiv, Plovdiv, Bulgaria; ^5^ Faculty of Health Sciences and Wellbeing, School of Medicine, University of Sunderland, Sunderland, United Kingdom; ^6^ Department of Hematology and Medical Oncology, Jena University Hospital, Jena, Germany; ^7^ Institute of Molecular Cell Biology, Center for Molecular Biomedicine Jena (CMB), Jena University Hospital, Jena, Germany

**Keywords:** metabolism, mitochondria, acute B-cell lymphoblastic leukaemia, glucocorticoid resistance, atovaquone

## Abstract

Childhood acute lymphoblastic leukaemia (cALL) accounts for about one third of all paediatric malignancies making it the most common cancer in children. Alterations in tumour cell metabolism were first described nearly a century ago and have been acknowledged as one of the key characteristics of cancers including cALL. Two of the backbone chemotherapeutic agents in the treatment of this disease, Glucocorticoids and L-asparaginase, are exerting their anti-leukaemic effects through targeting cell metabolism. Even though risk stratification and treatment regimens have improved cure rates to nearly 90%, prognosis for relapsed children remains poor. Therefore, new therapeutic approaches are urgently required. Atovaquone is a well-tolerated drug used in the clinic mainly against malaria. Being a ubiquinone analogue, this drug inhibits co-enzyme Q10 of the electron transport chain (ETC) affecting oxidative phosphorylation and cell metabolism. In this study we tested the effect of Atovaquone on cALL cells *in vitro*. Pharmacologically relevant concentrations of the inhibitor could effectively target mitochondrial respiration in both cALL cell lines (REH and Sup-B15) and primary patient samples. We found that Atovaquone leads to a marked decrease in basal respiration and ATP levels, as well as reduced proliferation, cell cycle arrest, and induction of apoptosis. Importantly, we observed an enhanced anti-leukaemic effect when Atovaquone was combined with the standard chemotherapeutic Idarubicin, or with Prednisolone in an *in vitro* model of Glucocorticoid resistance. Repurposing of this clinically approved inhibitor renders further investigations, but also presents opportunities for fast-track trials as a single agent or in combination with standard chemotherapeutics.

## Introduction

Altered cancer cell metabolism, described nearly a century ago by Warburg (1), has gone a long way from an intriguing scientific observation to a validated drug target in the clinic. Initially believed to serve as an alternative energy source for the rapidly dividing cancer cells, “Warburg’s effect” of oxidative glycolysis is now considered an effective means of metabolic rewiring which provides multiple precursors for key biosynthetic pathways (2–4). Excellent examples showing alterations in cell bioenergetics and the clinical use of certain metabolic vulnerabilities come from acute lymphoblastic leukaemia (ALL) (4).

Childhood ALL (cALL) accounts for about one third of all paediatric cancers. The disease is characterised by undifferentiated highly proliferative lymphoid cells of B-cell (~85%), T-cell (10–15%) or mixed lineage (<5%). The genetic and epigenetic aberrations underlying this malignancy are marked by great heterogeneity among patients with a recent study highlighting that there may be over 20 sub-types of B-cell cALL (5). Furthermore, there are a number of metabolic alterations in cALL present already at diagnosis. Some examples include aberrant transcriptional profile involving upregulation of glycolysis and downregulation of citric acid cycle genes (6), mutations in mitochondrial DNA affecting Oxidative Phorphorylation (OxPhos) (7), activation of the AKT pathway in T-ALL, which can cause upregulation of glycolysis and metabolic rewiring (8, 9), and others. Many of the standard chemotherapeutics can target cell metabolism. Glucocorticoids (GCs), the backbone of induction therapy, are effectively decreasing glucose uptake and shifting metabolism towards glutamine synthesis and fatty acid oxidation (10, 11). Anthracyclins inhibit topoisomerase II, but can also block Complex I of the electron transport chain (ETC) decreasing mitochondrial function (11, 12). L-Asparaginase is another critical part of standard treatment. It depletes extracellular asparagine levels, but also leads to glutamine deamination (13), glycolysis, and pyrimidine synthesis (14). Lastly, Methotrexate inhibits the folic acid cycle, thus the synthesis of nucleotides (15).

Refined risk group stratification and treatment regimens, including targeting of metabolic vulnerabilities in blast cells, have improved overall survival to 85–90% (16). Nevertheless, 5-year event-free survival after relapse for certain sub-groups of patients (*e.g*. with isolated bone marrow relapse, or deletion of *IKZF1*) may be less than 30% (17). Therefore, novel therapeutic approaches are urgently required especially for high risk groups, refractory disease, and relapsed patients.

Drug repurposing is a strategy whereby a drug, which is already approved for the treatment of a certain condition, is transferred into a different disease context. The advantage of this approach is saving considerable amount of time (at least 2–3 times quicker (18)) and money (3–10 times less on average (19)), ultimately translating into saving more lives faster and at a lower cost. Encouraging examples of drug repositioning already in use or in clinical trials range from Aspirin in colorectal cancer (20), Metformin (for diabetes) and Daunorubicin (antibiotic) for various malignancies (20, 21), retinoic acid (vitamin A) in acute promyelocytic leukaemia (APL) (22), a combination of retinoic acid and Tranylcypromine (TCP—anti-depressant) in acute myeloid leukaemia (23), and others.

Atovaquone (Ato) is an analogue of ubiquinone (the oxidised form of coenzyme Q10) making it an effective inhibitor of complex III of the electron transport chain (ETC) (24). Due to its high specificity and excellent tolerability Ato has been used against protozoa like *Plasmodium spp* and *Toxoplasma gondii*, turning it into a key component of standard anti-malarial therapy including in children (25). Interestingly, in recent years Ato has been highlighted as a potent anti-proliferative and apoptotic agent in different malignancies. For instance, Ato has proved effective *in vitro* in breast cancer (26), in pharyngeal and colorectal cancer cell lines in enhancing the effect of radiotherapy (27), in retinoblastoma cells (28), and in haematologic malignancies like chronic lymphoblastic leukaemia (29) and acute myeloid leukaemia (30). Nevertheless, the effect of Ato on cALL has not been studied.

Therefore, given the body of evidence in literature that cALL cells have alterations in their mitochondrial and metabolic function, together with encouraging results using Ato in other malignancies, the aim of our study was to investigate if Ato may be effective against cALL cells as well.

## Materials and Methods

### Cell Culture, Drug Treatments, Cell Viability, and Cell Counts

The childhood ALL cell line REH (harbouring a TEL-AML1 fusion, isolated after 1^st^ relapse) was kindly provided by Dr. Tino Schenk from the University Hospital of Jena (Germany), whilst Sup-B15 was purchased from DSMZ (Germany). Both cell lines were cultured in standard conditions—REH cells in RPMI with 10% foetal bovine serum (FBS) and 0.5% Penicillin–Streptomycin (Pen/Strep), and Sup-B15 (harbouring a BCR-ABL1 fusion, isolated after 2^nd^ relapse) in IMDM with 20% FBS and 0.5% Pen/Strep (all from PAN-Biotech, Germany). Atovaquone, Idarubicin, and Prednisolone (Cayman Chemical, USA, European Division in Estonia) were dissolved in DMSO (PAN-Biotech, Germany) to 20, 10, and 10 μM respectively. IC_50_ values were determined following drug treatment of 3 days and assessment of cell viability with MTT assay. All experiments were performed at least in biological duplicates and technical triplicates. Cell counts were determined using Luna II cell counter (Logosbio, South Korea).

### Establishment of Stable Prednisolone-Resistant Cells

To develop Prednisolone-resistant sub-clones of Sup-B15 cells, we used alternating cycles of drug treatment with increasing concentrations of Prednisolone by small increments followed by cell expansion. In brief, the IC_50_ concentration of Prednisolone for Sup-B15 was determined, and cells were treated with this concentration of 0.002 μM for 3 days. Surviving cells were centrifuged to eliminate dead cells, cell debris, and any remaining Prednisolone, washed with medium, and left to recover and expand in growth medium for 3–4 days. These cells were then treated with 1.5× IC_50_ of Prednisolone for another 3 days, then centrifuged, washed, and left in growth medium to expand again. The third cycle used 2× IC_50_ of Prednisolone, and every following cycle used increments of 30–50% higher concentration, compared to the previous cycle. Every four to five cycles Prednisolone was titrated to determine the shift in IC_50_ compared to the parental Sup-B15 cells. These cycles of treatment with increased concentrations of Prednisolone followed by cell recovery and expansion (>15 cycles) were repeated until we reached about 1,000 times resistance compared to parental Sup-B15 cells.

### Patient Samples

Bone marrow aspirates from patients were collected for standard diagnosis by flow cytometry. Cells from the remaining samples were isolated using Pancoll (PAN-Biotech) following the instructions by the manufacturer. At least 300,000 cells per well were plated for Atovaquone treatment, cultured for 3 days and analysed with the Seahorse XFp Instrument. Where possible, 30, 60, and 90 μM of Atovaquone were tested. For assessment of viability and the effect of Atovaquone and Idarubicin combinations, patient samples were seeded at ~50,000 cells per well in 96-well plates. All experiments with patient samples were performed in technical duplicates and triplicates where cell numbers allowed. Samples were taken after informed consent by the parents/guardians of the patients, and the study was approved by the Ethics Committee at the Medical University of Plovdiv.

### Seahorse XFp Analysis of Mitochondrial Function

For assessment of mitochondrial function we used Seahorse XFp analyser and Mito Stress Test kit (Agilent, USA). For this Mito Stress protocol, three mitochondrial inhibitors (all from Cayman Chemical, USA, European Division in Estonia) were injected consecutively onto the cells—Oligomycin (inhibitor of the ATP Synthetase) at 1 μM final concentration, FCCP (trifluoromethoxy carbonylcyanide phenylhydrazone—an uncoupler of the ETC) at 2 μM final concentration and Rotenone (inhibitor of Complex I of the ETC) at 0.5 μM final concentration. This allowed the assessment of several parameters including basal and maximal respiration, spare respiratory capacity, ATP levels, and others. B-cell ALL cells were grown in 12- or 24-well plates with or without Atovaquone for 72 h. Cells were then centrifuged and washed twice with Seahorse-compatible bicarbonate buffer-free RPMI (AppliChem, Germany) supplemented with Pyruvate (1 mM) with adjusted final pH of 7.2–7.4. Cells were then counted with Luna II cell counter (LogosBio, South Korea) and seeded at 100–300,000/well in Seahorse microplates (Agilent, USA), which were pre-coated with Poly-D-Lysine at 1 mg/ml for 30 min. Seahorse oxygen consumption rate (OCR) data for each experiment was normalised to total live cells per well counted with Luna II cell counter (LogosBio, South Korea) using Seahorse Wave Desktop Software v 2.6.0 (Agilent, USA). Data analysis was then performed using the XF Mito Stress Report Generator within Wave Desktop Software.

### Cell Cycle and Apoptosis Assays

Cell cycle analysis and Annexin V/PI assay for apoptosis were carried out on a Guava^®^ Muse^®^ Cell Analyser (Luminex, USA) using the respective standard kits and protocols optimised by Luminex.

### RNA-Sequencing

RNA extraction from control untreated and Ato-treated (30 μM) samples in biological triplicates was carried out on day 3 after treatment from ~1 million pelleted cells per condition with Qiagen RNeasy kit (Qiagen, USA) according to the manufacturer’s recommendations. Initial sample concentration and quality was assessed by Nanodrop (Thermo, USA). Samples were then sent to Novogene UK for further QC, reverse transcription, library preparation, and sequencing. In brief, NEBNext^®^ UltraTM RNA Library Prep Kit for Illumina^®^ (NEB, USA) was used for library preparation with AMPure XP beads (Beckman Coulter, Beverly, USA) for size selection and PCR purification steps, both performed as instructed by the manufacturers. Finally, sequencing was performed on an Illumina Sequencer generating >20M pair-end clean reads per sample.

### Synergy Testing

Analysis of drug combinations was carried out with SynergyFinder (31). The zero interaction potency (ZIP) model (32) was chosen as the default parameter in SynergyFinder (together with four parameter logistic regression algorithm, LL4, for curve fitting) to calculate *δ* (delta) scores/synergy scores for the interaction between Atovaquone and Idarubicin. The reference point in the ZIP model is zero (*i.e. δ* = 0), which implies zero interaction between the two drugs. Synergy scores lower than zero (*i.e*. δ < 0) imply a likely antagonistic interaction (*δ* <−10 is considered most likely antagonistic), whilst *δ* > 0 shows additivity or synergy between the two tested drugs (δ > 10 would be interpreted as most likely synergistic) (32).

### Statistical Analysis

Sequencing quality control, mapping, quantification, and differential gene expression analysis were also performed by Novogene using HISAT2 software, RPKM calculation for each gene, DESeq2 and EdgeR package in R to generate lists with differentially expressed genes between Atovaquone treatment and control samples. Adjusted *p*-value (≤0.05) and fold change (FC) of ≥1.3 and ≤−1.3 were used as thresholds to create the gene lists necessary for downstream analysis. Gene Ontology (GO) analysis was carried out by BinGO (33) in Cytoscape Software (34) where statistically significantly enriched nodes (*p*- ≤0.05) have been colour-coded (yellow for 5 × 10^−2^ to dark orange for 5 × 10^−7^). Gene set enrichment analysis (GSEA) was performed from generated lists with differentially expressed genes as described above using the GSEA software by the Broad Institute (35) (standard settings of 1,000 permutations, permutation type—gene_set, and Molecular Signature Database gene sets—H: Hallmark, C2: Curated gene sets, C5: ontology gene sets, C6: oncogenic signatures gene sets). The default FDR cut-off is 0.25; however, only gene sets with FDR ≤0.05 were plotted. GraphPad Prism (v. 8) was used for figure preparation and statistical analysis (parametric unpaired Student’s *t*-test for Seahorse and MTT data and paired Student’s *t*-test for patient samples’ standard cut-offs of *p*- ≤0.05).

## Results

### Atovaquone Demonstrates Anti-Leukaemic Properties *In Vitro* Through Inhibition of Mitochondrial Respiration

**Figure 1:**
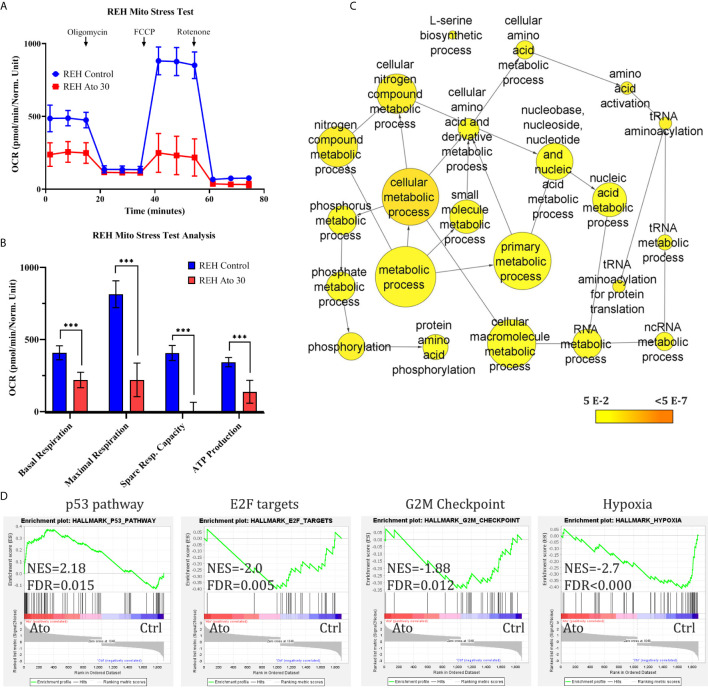
Atovaquone treatment targets cell mithochondrial respiration and activates apoptosis and cell cycle arrest in REH cells. REH cells were treated with IC_50_ concentrations of Atovaquone (30 µM) for 3 days and compared to untreated control cells. **(A)** Their mitochondrial function was analysed with Seahorse XFp Analyser Instrument using the Mito Stress Test (Agilent, USA). **(B)** The results of the test showed reduction in basal and maximal respiration, spare respiratory capacity and ATP production after treatment with Ato. **(C)** Control and Ato-treated REH cells were collected on day 3 and RNA-sequencing was carried out. Gene ontology (GO) analysis with BinGO in Cytoscape showed a cluster of statistically significantly represented nodes related to metabolic processes as shown (statistically not significant nodes were removed from the cluster). Scale bar (yellow-orange) depicts *p*-values as annotated. **(D)** Gene set enrichment analysis (GSEA) was also performed with the GSEA software by the Broad Institute. The analysis demonstrates strong statistically significant (FDR q-value <0.05) enrichment for the shown gene sets. Error bars represent mean with SD from biological triplicates in technical duplicates. ***p < 0.0005, Student’s *t*-test. NES, normalised enrichment score; FDR, false discovery rate q-value.

The anti-proliferative effect of Atovaquone on REH cells was investigated first. We found that at IC_50_ concentration (**Supplementary Figure 1A**) Atovaquone exerts its anti-leukaemic effect through both deceleration of the cell cycle (53.4 ± 7.7% in G1-phase in control cells *vs* 67.6 ± 3.5% in Ato-treated cells, *p* = 0.044, as well as 29.6 ± 6.3% of control cells in S-phase *vs* 19.3 ± 1.7% in Ato-treated cells, *p* = 0.05, **Supplementary Figure 1B**). Ato also increased the number of apoptotic cells (19.8 ± 2.9% apoptotic cells in control *vs* 30.2 ± 6.6% in Ato-treated cells, *p* = 0.005, **Supplementary Figure 1C**). In order to elucidate the potential mechanism of action of Ato. we examined the mitochondrial function of the cells following treatment with the drug for three days (**Figure 1A**). We found that Ato treatment led to a ~two-fold reduction of basal respiration (from 407.6 ± 48.8 to 219.4 ± 53.9, *p* < 0.0001) and ~3.7-fold decrease in maximal respiration (from 813.8 ± 93.1 to 220.2 ± 116, *p* < 0.0001). It nearly completely abolished spare respiratory capacity (from 406.2 ± 52.1 to 0.8 ± 63.6, *p* < 0.0001) and decreased ATP production by more than half (from 343 ± 32.3 to 137.6 ± 79.6, *p* = 0.0002) (**Figure 1B** and **Supplementary Table 1**).

RNA-seq (**Figures 1C, D**, **Supplementary Figures 2 and 3**, and **Supplementary Table 2**) aiming to elucidate the effect of Ato on REH cells showed that this drug changed the expression of nearly 2,000 genes compared to untreated cells (with cut-offs of adjusted *p*-value <0.05, FC >1.3). Gene ontology (GO) analysis revealed enrichment for cell metabolic processes (**Figure 1C**), apoptosis, cell cycle arrest, and a small cluster of oxidative stress genes (**Supplementary Figures 2A–C**), which is another confirmation of the phenotypic effects we observed. Further Gene Set Enrichment Analysis (GSEA) demonstrated—upregulation of the p53 pathway, downregulation of E2F, and G2M checkpoint (both related to cell cycle control), and of hypoxia gene sets (**Figure 1D**). We further found that Ato treatment may enhance fatty acid metabolism and, interestingly, downregulate RNA metabolic processes (**Supplementary Figure 3**).

**Figure 2:**
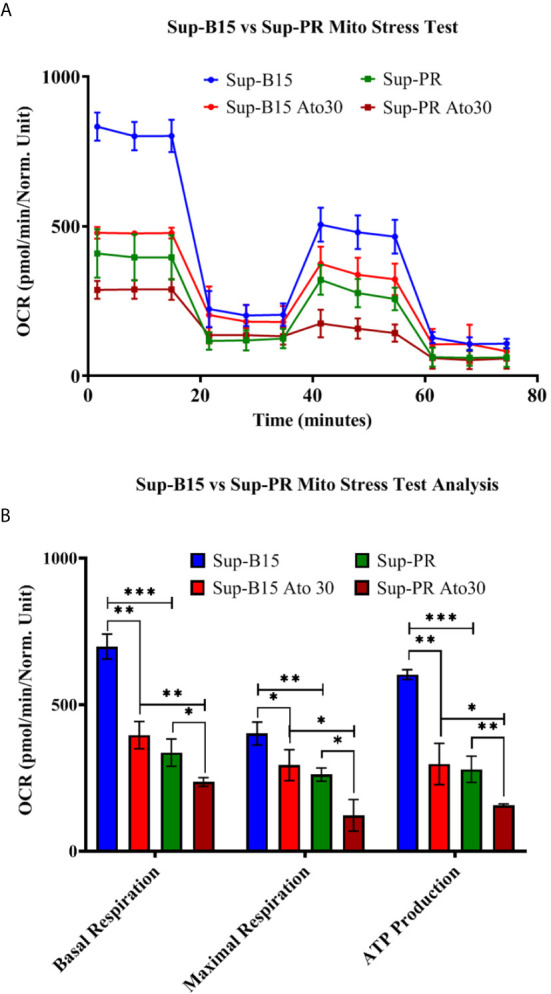
Atovaquone decreases mitochondrial respiration and ATP production in Sup-B15 cells and Prednisolone-resistant Sup-PR cells. Sup-B15 and Sup-PR cells in biological duplicates were treated with 30 µM of Ato for 3 days and compared to control untreated cells. **(A)** Sup-B15 cells and Sup-PR cells were assayed with Mito Stress Test on Seahorse XFp Analyser (Agilent, USA) and **(B)** were analysed for changes in mitochondrial respiration and ATP production. Error bars represent mean with SD from biological duplicates in technical duplicates. *p ≤ 0.05, **p ≤ 0.005, ***p < 0.0005, Student’s *t*-test.

Another B-cell precursor cALL cell line—Sup-B15 was analysed as well. We also made use of a Glucocorticoid-resistant sub-clone of Sup-B15 referred to as “Sup-PR” that was established in our laboratory (*Methods* and **Supplementary Figure 1D**). We observed that Ato had similar IC_50_ values as in REH cells (~30 μM, **Supplementary Figure 1E**) and elicited very similar changes in mitochondrial respiration and ATP production in both Sup-B15 and Sup-PR cells (**Figures 2A, B**). Of note, GC-resistance of around 1,000 times did not change the response to Ato, and we found significant reduction of basal and maximal respiration, as well as of ATP production in Sup-PR cells (**Supplementary Table 3** and **Figure 2B**).

### Atovaquone Enhances the Effect of Standard Chemotherapeutics and Resensitises Resistant Cells to Glucocorticoids

**Figure 3:**
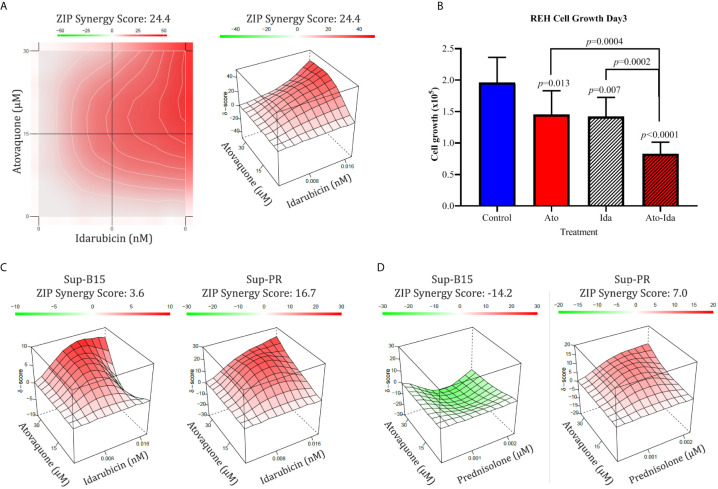
Combination of Atovaquone with standard chemotherapeutics proves effective in REH Prednisolone resistant Sup-PR cells. **(A)** REH cells and **(C)** Sup-B15 and Sup-PR cells were treated with combination of IC_50_ and IC_25_ concentrations of Atovaquone and Idarubicin in triplicates for three days and assessed by MTT test. The resulting cell viability matrix was tested by SynergyFinder. The data show % inhibition based on percentage viable cells (left) and the calculated synergy score (right). **(B)** Cell growth of REH cells treated as shown was assessed by cell counting with Luna II Cell Counter (LogosBio, South Korea). Cell numbers were plotted as labelled in the figure. Error bars represent SD of the mean for >6 separate counts from triplicates. p-values were calculated with, Student’s *t*-test. **(D)** Sup-B15 and Sup-PR cells were treated with IC_50_ and IC_25_ concentrations of Atovaquone and Prednisolone as single drugs and in combinations. Triplicates were analysed on day 3 by MTT and synergy scores were calculated by SynergyFinder as described above.

We also investigated if Ato may enhance the action of standard chemotherapeutics known to affect different aspects of cell metabolism. Thereby, we examined combinations with several chemotherapeutics which are part of the standard treatment regimen such as Methotrexate (targeting folic acid metabolism), Idarubicin (topoisomerase II inhibitor and inhibitor of complex I of the ETC), and Prednisolone (targeting glycolysis). We found marked synergistic effect (ZIP synergy score of 24.4) in REH cells only when Ato was in combination with Idarubicin (**Figure 3A** and **Supplementary Figure 1F** for IC_50_ values) and not in any of the other two combinations (data not shown). Furthermore, this synergy had a marked effect on cell proliferation as well (from 1.96 ± 0.4 × 10^5^ live control cells on day 3 to 0.83 ± 0.19 × 10^5^ live cells treated with Ato and Idarubicin, p < 0.0001, **Figure 3B** and **Supplementary Table 4**). We also investigated if this combination would work in Sup-B15 and Sup-PR cells (**Supplementary Figure 1F** for IC_50_ values). Interestingly, whilst the two drugs demonstrated only additive effect on Sup-B15 cells (**Figure 3C**, synergy score of 3.6), Sup-PR proved more sensitive to this double treatment with a synergy score of 16.7 (**Figure 3C**).

Glucocorticoid (GC) resistance is believed to be one of the major causes of relapse in the clinic. Since we found that Sup-PR cells remain sensitive to Ato, we then investigated if Ato can re-sensitise these cells to Prednisolone. Therefore, we examined Ato-Pred double combination in Sup-B15 and Sup-PR cells. What we discovered was that whilst this combination in the parental Sup-B15 cells may even prove to be counterproductive (antagonistic ZIP score of −14.2—**Figure 3D**), this treatment had an additive effect on Sup-PR cells (score of 7—**Figure 3D**).

### Validation of the Effect of Atovaquone in Patient Samples

**Figure 4:**
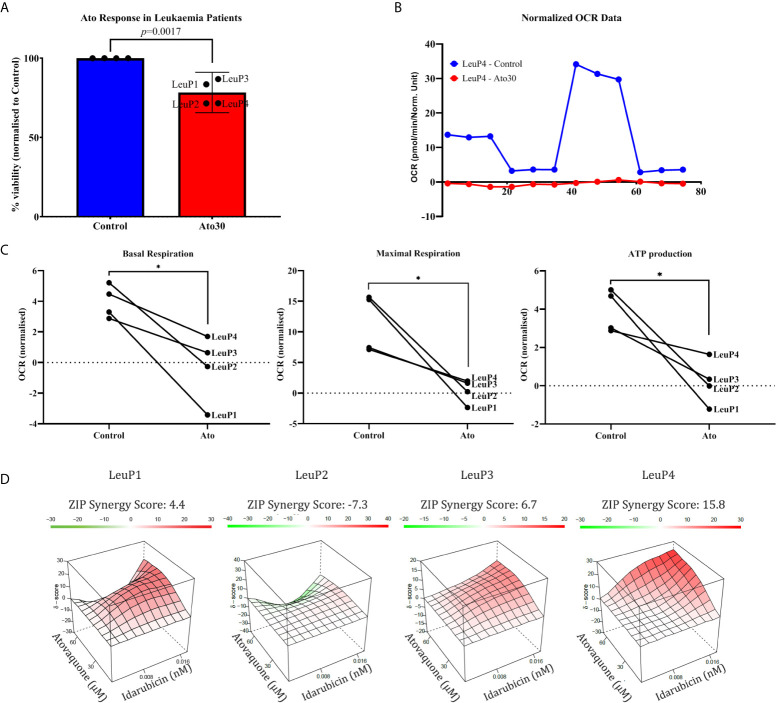
Atovaquone shows efficacy in primary cALL lymphoblasts. **(A)** Isolated patient cells were cultured for 3 days with 30 μM Atovaquone, and cell viability was assessed by MTT assay. Percentage live cells normalised to control (100% viable) were calculated for each sample and plotted as shown. **(B)** A representative Mito Stress Test graph from patient #2 showing the effect of 30 μM Atovaquone on mitochondrial respiration. **(C)** Analysis of Mito Stress Tests for basal and maximal respiration as well as for ATP production as shown. The data from the highest tested concentration in each patient sample (60 μM for LeuP1 and 90 μM for LeuP2, 3 and 4) was analysed.*p ≤ 0.05, paired Student’s *t*-test. **(D)** SynergyFinder analysis of the combinatorial effect of Atovaquone (30 and 60 μM) and Idarubicin (0.0008 and 0.0016 μM) in four patient samples as annotated. LeuP, Leukaemia patient.

Lastly, we attempted to validate our findings in a small number of patient samples available from the Oncohaematology Unit at the University Clinic of Paediatrics (**Figures 4A–D** and **Supplementary Table 5**). Importantly, we found that Ato is capable of reducing growth in primary lymphoblasts at 30 μM concentrations (**Figure 4A**), which are lower than the maximum achievable plasma levels *in vivo* (see *Discussion*). Furthermore, we validated that Ato is capable of targeting mitochondrial respiration and ATP production in patient samples (**Figures 4B, C** and **Supplementary Table 6**). Lastly, we investigated the effect of combining Ato and Idarubicin in these samples. Interestingly, we found that this combination elicits a more variable response between the samples ranging from antagonistic (in one sample) to additive (in two samples), and synergistic (in one sample) (**Figure 4D**).

## Discussion

In summary, we have investigated the anti-proliferative activity of Atovaquone on two established B-cell precursor cALL cell lines—REH and Sup-B15, as well as on a Prednisolone-resistant sub-clone of Sup-B15. We found that Ato elicited a very similar effect on mitochondrial respiration and ATP production in all three cell lines at concentrations of ~30 μM. These findings are in line with previous *in vitro* studies [10 μM in breast cancer (26) and cervical cancer cells (36), 30 μM in colorectal, pharyngeal and lung cancer cell lines (27, 37), ~15–50 μM for glioblastoma cells (38), 20 μM in retinoblastoma (28) and thyroid cancer cells (39)]. In particular, Ato reduced the levels of basal and maximal respiration, decreased the respiratory capacity of the cells and ATP production as measured by the Seahorse Mito Stress test. Even if we could carry out these experiments on a limited number of patient samples, we observed that Ato can induce similar changes of mitochondrial respiration in primary lymphoblasts as well.

GSEA and GO analyses in REH cells confirmed some of the phenotypic observations in our study—alterations in cell metabolism, induction of apoptosis, and cell cycle arrest (**Figure 1** and **Supplementary Figure 2**), and pointed to other interesting concomitant changes that may be due to Ato. Several papers from the past 5 years have investigated the anti-cancer effect of Ato. Most of them have focussed on inhibition of OxPhos, which we confirm herein as well, whilst two publications show that Ato may work through targeting the AKT–mTOR pathway (28). The interplay between mTOR complex 1 (mTORC1) and mitochondrial function and cell metabolism is well-documented (40). Activation of the AKT–mTOR pathway, which is altered in up to one third of children with T-ALL (41), may lead to considerable changes in cell metabolism and upregulation of glycolysis (4, 42). Investigating the potential effect of Ato on this pathway was beyond the scope of our study. Nevertheless, one of the most downregulated genes we found (log2 = −2.1, adjusted *p*-value = 0.0018) is *PTEN* and GSEA from our RNA-seq showed significant and strong downregulation of mTORC1 genes (**Supplementary Table 2** and **Supplementary Figure 3**). Of note, there are links between mTORC1 and the MYC oncoprotein, as well as between overexpression of *MYC* and shifts in cALL metabolism (9). Therefore, it is noteworthy that our GSEA also revealed downregulation of MYC targets in Ato-treated cells (**Supplementary Figure 3**).

Ato has been shown to alleviate hypoxia and sensitise cancer cells to radiotherapy (27). Our GSEA analysis highlights alterations of hypoxia as one of the top hits and indeed we see downregulation of the direct HIF1-*α* target *VEGFα* after treatment (**Supplementary Table 2**). There is evidence that radiation therapy for high-risk T-ALL patients may reduce rates of relapse (43). Given that hypoxia is a major factor for the efficacy of radiation therapy, that Ato reduces hypoxia in pre-clinical models (27, 44) and in our gene set enrichment analysis (**Figure 1**), Ato treatment may be worthy of further investigation in this clinical context of B-cell cALL too.

The combination of Ato with Idarubicin is another interesting aspect of our study. There is an independent confirmation of the synergy between Ato and anthracyclins coming from work on thyroid cancer cells. Lv and colleagues found that Ato and Doxorubicin are an effective strategy working through ETC and consequent STAT3 inhibition (39). Of note, Ato and Idarubicin were most effective in REH cells and Sup-PR cells, both of which are proliferating faster than Sup-B15. Therefore, it may be interesting to test this combination on matched patient samples at diagnosis and relapse, but this is beyond the scope of our current work.

GC-resistance is believed to be the main cause of drug-failure and relapse in cALL (45). Therefore, it was interesting to find that Sup-PR cells remain sensitive to Ato and that there was an additive effect of Ato and Prednisolone in this GC-resistant line, but not in the parental cells. Investigating the molecular mechanisms of resistance (*e.g.* why Sup-PR cells have significantly lower oxygen consumption rates than Sup-B15 cells—**Figure 2**) and of re-sensitisation to Prednisolone by Ato was another important aspect which fell out of the scope of our study. However, the effectiveness of Ato alone and in combination with Idarubicin or Prednisolone in GC-resistant cells is worthy of further pre-clinical investigations with matched samples and may add another context to a potential clinical trial.

Importantly, there are several key points related to the patient samples’ data and the potential clinical applicability of this study that need to be addressed. Due to the small number of available patient samples, which all belong to the most common type of cALL and its most frequent molecular subtype, one of the main limitations of our work is the lack of depth and representativity of different entities within this heterogeneous disease. Even though the experimental data with these four patient samples cannot be over-interpreted and directly translated to the clinic, we show as a proof-of-concept that Ato can hit its target in primary lymphoblasts as well. We observe significant decrease in mitochondrial respiration and ATP production (**Figure 4B** and **Supplementary Table 6**), similarly to what we have in REH, Sup-B15, and Sup-PR cells. Nevertheless, the effect of Ato on cell viability in the patient samples (**Figure 4A**) is not as prominent as in the cell lines. This modest decrease in cell proliferation, however, may be due to either sub-optimal concentrations of Ato or to the very slow cell division of cultured primary cells, which would not allow for the detection of potential cell cycle arrest (as seen in the fast proliferating REH cells) and would also account for the low OCR readings especially after Ato treatment. We also found that the combination of Ato and Idarubicin in patient samples elicits a range of responses—from antagonistic to synergistic. Besides potential differences in proliferation rates *ex vivo*, which may account for the lack of uniformity in the response to Ato and Idarubicin, the small number of patient samples does not allow for any speculations other than that there may be unidentified molecular and/or cytogenetic factors which may determine either advantageous or adverse results of the double treatments. Therefore, a larger cohort of patients including relapsed samples may be worth testing.

Lastly, the IC_50_ concentration we found for our cell lines of 30 μM (~11 ug/ml) (**Supplementary Figures 1A, E** **)** and the concentrations of maximum 90 μM (~33 ug/ml) that we managed to test on some of the patient samples are within the clinically recorded levels for Ato. The steady-state plasma concentration in children achieved by daily administration of 30 mg/kg for 12 days is 37.1 ± 10.9 ug/ml (as described in the prescribing information for Mepron^®^). Other literature data suggests similar values—Cmax and Cmin of respectively 40.5 ± 12.2 and 34.0 ± 11.7 ug/ml (of 30 mg/kg daily) (46). Furthermore, there is an interesting finding coming from a retrospective study of over 200 patients with AML who had undergone bone marrow transplantation. What Xiang et al. found was that administration of Ato for nearly two months as a part of standard anti-infection prophylaxis led to significant reduction of relapse rates (13 *vs* 23%, *p* = 0.039) (30). Therefore, considering the number of robust pre-clinical studies in other cancers, the good safety data for Ato and the clear effect on proliferation due to ETC inhibition, it is tempting to speculate that Ato would be worthy of further pre-clinical *in vivo* investigation and perhaps clinical trials as the ongoing one on Ato and radiation therapy (NCT02628080).

In conclusion, we present novel data demonstrating the anti-leukaemic effect of Atovaquone, the mechanism of action of the drug and the concomitant gene expression changes that may underpin the phenotypic changes observed. Furthermore, our results suggest that this FDA-approved ETC inhibitor may have additive or synergistic effect together with Idarubicin. GC-resistant cells may be re-sensitised to Prednisolone when treated with Atovaquone. Therefore, the findings of this study may present a promising new therapeutic approach targeting cell metabolism, which may be worthy of further investigation.

## Data Availability Statement

The original contributions presented in the study are publicly available. This data can be found here: https://www.ncbi.nlm.nih.gov/geo/query/acc.cgi?acc=GSE161959.

## Ethics Statement

The studies involving human participants were reviewed and approved by the Ethics committee at Medical University of Plovdiv. Written informed consent to participate in this study was provided by the participants’ legal guardian/next of kin.

## Author Contributions

YS, TI, TS, KP, and VS designed the study. YS, TI, HB, and KG developed the methodology. YS, TI, TS, KP, and VS analysed the data. YS, VS, TS, and KP wrote and edited the manuscript. All authors contributed to the article and approved the submitted version.

## Funding

This project was funded by the Bulgarian National Science Fund (project No. KP-06-M21/9 /19.12.18), and supported by the National University Complex for Biomedical and Applied Research with participation in BBMRI-ERIC, NUCBPI-BBMRI.BG, (contracts No. D01-285/ 17.12.2019 and DO1-395/ 18.12.2020) within the National Road Map For Research Infrastructure.

## Conflict of Interest

The authors declare that the research was conducted in the absence of any commercial or financial relationships that could be construed as a potential conflict of interest.

## References

[1] Warburg O Wind F Negelein E. The Metabolism of Tumors in the Body. J Gen Physiol (1927) 8:519–30. 10.1085/jgp.8.6.519 PMC2140820 19872213

[2] DeBerardinis RJ Chandel NS. Fundamentals of cancer metabolism. Sci Adv (2016) 2:e1600200. 10.1126/sciadv.1600200 27386546 PMC4928883

[3] Luengo A Gui DY Vander Heiden MG. Targeting Metabolism for Cancer Therapy. Cell Chem Biol (2017) 24:1161–80. 10.1016/j.chembiol.2017.08.028 PMC5744685 28938091

[4] Sbirkov Y Burnusuzov H Sarafian V. Metabolic reprogramming in childhood acute lymphoblastic leukemia. Pediatr Blood Cancer (2020) 67:e28255. 10.1002/pbc.28255 32293782

[5] Gu Z Churchman ML Roberts KG Moore I Zhou X Nakitandwe J. PAX5-driven subtypes of B-progenitor acute lymphoblastic leukemia. Nat Genet (2019) 51:296–307. 10.1038/s41588-018-0315-5 30643249 PMC6525306

[6] Boag JM Beesley AH Firth MJ Freitas JR Ford J Hoffmann K. Altered glucose metabolism in childhood pre-B acute lymphoblastic leukaemia. Leukemia (2006) 20:1731–7. 10.1038/sj.leu.2404365 17041637

[7] Kodron A Ghanim M Krawczyk KK Stelmaszczyk–Emmel A Tonska K Demkow U. Mitochondrial DNA in pediatric leukemia patients. Acta Biochim Pol (2017) 64:183–7. 10.18388/abp.2016_1444 28284021

[8] Jenkinson S Koo K Mansour MR Goulden N Vora A Mitchell C. Impact of NOTCH1/FBXW7 mutations on outcome in pediatric T-cell acute lymphoblastic leukemia patients treated on the MRC UKALL 2003 trial. Leukemia (2013) 27:41–7. 10.1038/leu.2012.176 22814294

[9] Herranz D Ambesi–Impiombato A Sudderth J Sanchez–Martin M Belver L Tosello V. Metabolic reprogramming induces resistance to anti-NOTCH1 therapies in T cell acute lymphoblastic leukemia. Nat Med (2015) 21:1182–9. 10.1038/nm.3955 PMC4598309 26390244

[10] Dyczynski M Vesterlund M Bjorklund AC Zachariadis V Janssen J Gallart–Ayala H. Metabolic reprogramming of acute lymphoblastic leukemia cells in response to glucocorticoid treatment. Cell Death Dis (2018) 9:846. 10.1038/s41419-018-0625-7 30154400 PMC6113325

[11] Aoki S Morita M Hirao T Yamaguchi M Shiratori R Kikuya M. Shift in energy metabolism caused by glucocorticoids enhances the effect of cytotoxic anti-cancer drugs against acute lymphoblastic leukemia cells. Oncotarget (2017) 8:94271–85. 10.18632/oncotarget.21689 PMC5706873 29212227

[12] Yadav N Kumar S Marlowe T Chaudhary AK Kumar R Wang J. Oxidative phosphorylation-dependent regulation of cancer cell apoptosis in response to anticancer agents. Cell Death Dis (2015) 6:e1969. 10.1038/cddis.2015.305 26539916 PMC4670921

[13] Panosyan EH Grigoryan RS Avramis IA Seibel NL Gaynon PS Siegel SE. Deamination of glutamine is a prerequisite for optimal asparagine deamination by asparaginases *in vivo* (CCG-1961). Anticancer Res (2004) 24:1121–5. 15154634

[14] Hermanova I Arruabarrena–Aristorena A Valis K Nuskova H Alberich–Jorda M Fiser K. Pharmacological inhibition of fatty-acid oxidation synergistically enhances the effect of l-asparaginase in childhood ALL cells. Leukemia (2016) 30:209–18. 10.1038/leu.2015.213 26239197

[15] Umerez M Gutierrez-Camino A Munoz-Maldonado C Martin-Guerrero I Garcia-Orad A. MTHFR polymorphisms in childhood acute lymphoblastic leukemia: influence on methotrexate therapy. Pharmgenomics Pers Med (2017) 10:69–78. 10.2147/PGPM.S107047 28392709 PMC5376125

[16] Kato M Manabe A. Treatment and biology of pediatric acute lymphoblastic leukemia. Pediatr Int (2018) 60:4–12. 10.1111/ped.13457 29143423

[17] Bhojwani D Pui CH. Relapsed childhood acute lymphoblastic leukaemia. Lancet Oncol (2013) 14:e205–217. 10.1016/S1470-2045(12)70580-6 23639321

[18] Ashburn TT Thor KB. Drug repositioning: identifying and developing new uses for existing drugs. Nat Rev Drug Discov (2004) 3:673–83. 10.1038/nrd1468 15286734

[19] Pushpakom S Iorio F Eyers PA Escott KJ Hopper S Wells A. Drug repurposing: progress, challenges and recommendations. Nat Rev Drug Discov (2019) 18:41–58. 10.1038/nrd.2018.168 30310233

[20] Nowak-Sliwinska P Scapozza L Ruiz i Altaba A. Drug repurposing in oncology: Compounds, pathways, phenotypes and computational approaches for colorectal cancer. Biochim Biophys Acta Rev Cancer (2019) 1871:434–54. 10.1016/j.bbcan.2019.04.005 PMC6528778 31034926

[21] Rudrapal M Khairnar SJ Jadhav AG. Drug Repurposing (DR): An Emerging Approach in Drug Discovery. IntechOpen (2020) 1–20. 10.5772/intechopen.93193

[22] Degos L Wang ZY. All trans retinoic acid in acute promyelocytic leukemia. Oncogene (2001) 20:7140–5. 10.1038/sj.onc.1204763 11704842

[23] Schenk T Chen WC Gollner S Howell L Jin L Hebestreit K. Inhibition of the LSD1 (KDM1A) demethylase reactivates the all-trans-retinoic acid differentiation pathway in acute myeloid leukemia. Nat Med (2012) 18:605–11. 10.1038/nm.2661 PMC3539284 22406747

[24] Haile LG Flaherty JF. Atovaquone: a review. Ann Pharmacother (1993) 27:1488–94. 10.1177/106002809302701215 8305784

[25] Nixon GL Moss DM Shone AE Lalloo DG Fisher N O'Neill PM. Antimalarial pharmacology and therapeutics of atovaquone. J Antimicrob Chemother (2013) 68:977–85. 10.1093/jac/dks504 PMC4344550 23292347

[26] Fiorillo M Lamb R Tanowitz HB Mutti L Krstic–Demonacos M Cappello AR. Repurposing atovaquone: targeting mitochondrial complex III and OXPHOS to eradicate cancer stem cells. Oncotarget (2016) 7:34084–99. 10.18632/oncotarget.9122 PMC5085139 27136895

[27] Ashton TM Fokas E Kunz–Schughart LA Folkes LK Anbalagan S Huether M. The anti-malarial atovaquone increases radiosensitivity by alleviating tumour hypoxia. Nat Commun (2016) 7:12308. 10.1038/ncomms12308 27453292 PMC4962491

[28] Ke F Yu J Chen W Si X Li X Yang F. The anti-malarial atovaquone selectively increases chemosensitivity in retinoblastoma *via* mitochondrial dysfunction-dependent oxidative damage and Akt/AMPK/mTOR inhibition. Biochem Biophys Res Commun (2018) 504:374–9. 10.1016/j.bbrc.2018.06.049 29902460

[29] Das S Dielschneider R Chanas-LaRue A Johnston JB Gibson SB. Antimalarial drugs trigger lysosome-mediated cell death in chronic lymphocytic leukemia (CLL) cells. Leuk Res (2018) 70:79–86. 10.1016/j.leukres.2018.06.005 29902707

[30] Xiang M Kim H Ho VT Walker SR Bar–Natan M Anahtar M. Gene expression-based discovery of atovaquone as a STAT3 inhibitor and anticancer agent. Blood (2016) 128:1845–53. 10.1182/blood-2015-07-660506 PMC5054697 27531676

[31] Ianevski A Giri AK Aittokallio T. SynergyFinder 2.0: visual analytics of multi-drug combination synergies. Nucleic Acids Res (2020) 48:W488–93. 10.1093/nar/gkaa216 PMC7319457 32246720

[32] Yadav B Wennerberg K Aittokallio T Tang J. Searching for Drug Synergy in Complex Dose-Response Landscapes Using an Interaction Potency Model. Comput Struct Biotechnol J (2015) 13:504–13. 10.1016/j.csbj.2015.09.001 PMC4759128 26949479

[33] Maere S Heymans K Kuiper M. BiNGO: a Cytoscape plugin to assess overrepresentation of gene ontology categories in biological networks. Bioinformatics (2005) 21:3448–9. 10.1093/bioinformatics/bti551 15972284

[34] Shannon P Markiel A Ozier O Baliga NS Wang JT Ramage D. Cytoscape: a software environment for integrated models of biomolecular interaction networks. Genome Res (2003) 13:2498–504. 10.1101/gr.1239303 PMC403769 14597658

[35] Subramanian A Tamayo P Mootha VK Mukherjee S Ebert BL Gillette MA. Gene set enrichment analysis: a knowledge-based approach for interpreting genome-wide expression profiles. Proc Natl Acad Sci U S A (2005) 102:15545–50. 10.1073/pnas.0506580102 PMC1239896 16199517

[36] Tian S Chen H Tan W. Targeting mitochondrial respiration as a therapeutic strategy for cervical cancer. Biochem Biophys Res Commun (2018) 499:1019–24. 10.1016/j.bbrc.2018.04.042 29630860

[37] Coates JTT Rodriguez–Berriguete G Puliyadi R Ashton T Prevo R Wing A. The anti-malarial drug atovaquone potentiates platinum-mediated cancer cell death by increasing oxidative stress. Cell Death Discov (2020) 6:110. 10.1038/s41420-020-00343-6 33133645 PMC7591508

[38] Takabe H Warnken ZN Zhang Y Davis DA Smyth HDC Kuhn JG. A Repurposed Drug for Brain Cancer: Enhanced Atovaquone Amorphous Solid Dispersion by Combining a Spontaneously Emulsifying Component with a Polymer Carrier. Pharmaceutics (2018) 10(2):60. 10.3390/pharmaceutics10020060 PMC6027483 29783757

[39] Lv Z Yan X Lu L Su C He Y. Atovaquone enhances doxorubicin’s efficacy *via* inhibiting mitochondrial respiration and STAT3 in aggressive thyroid cancer. J Bioenerg Biomembr (2018) 50:263–70. 10.1007/s10863-018-9755-y 29687367

[40] de la Cruz Lopez KG Toledo Guzman ME Sanchez EO Garcia Carranca A. mTORC1 as a Regulator of Mitochondrial Functions and a Therapeutic Target in Cancer. Front Oncol (2019) 9:1373. 10.3389/fonc.2019.01373 31921637 PMC6923780

[41] Zuurbier L Petricoin EF 3rd Vuerhard MJ Calvert V Kooi C Buijs–Gladdines JG. The significance of PTEN and AKT aberrations in pediatric T-cell acute lymphoblastic leukemia. Haematologica (2012) 97:1405–13. 10.3324/haematol.2011.059030 PMC3436243 22491738

[42] Elstrom RL Bauer DE Buzzai M Karnauskas R Harris MH Plas DR. Akt stimulates aerobic glycolysis in cancer cells. Cancer Res (2004) 64:3892–9. 10.1158/0008-5472.CAN-03-2904 15172999

[43] Kassick M Koffer PP Kinsella TJ. Trends in the Use of Radiation Therapy for Pediatric T-Cell Acute Lymphoblastic Leukemia and Impact on Survival: A Population-Based Analysis. Int J Radiat Oncol (2016) 96:E553–554. 10.1016/j.ijrobp.2016.06.2014

[44] Ashton TM McKenna WG Kunz-Schughart LA Higgins GS. Oxidative Phosphorylation as an Emerging Target in Cancer Therapy. Clin Cancer Res (2018) 24:2482–90. 10.1158/1078-0432.CCR-17-3070 29420223

[45] Goto H. Childhood relapsed acute lymphoblastic leukemia: Biology and recent treatment progress. Pediatr Int (2015) 57:1059–66. 10.1111/ped.12837 26455582

[46] Hughes W Dorenbaum A Yogev R Beauchamp B Xu J McNamara J. Phase I safety and pharmacokinetics study of micronized atovaquone in human immunodeficiency virus-infected infants and children. Pediatric AIDS Clinical Trials Group. Antimicrob Agents Chemother (1998) 42:1315–8. 10.1128/AAC.42.6.1315 PMC105594 9624466

