# Supplementary material for: The Protozoan Inhibitor Atovaquone Affects Mitochondrial Respiration and Shows In Vitro Efficacy Against Glucocorticoid-Resistant Cells in Childhood B-Cell Acute Lymphoblastic Leukaemia
Source: Front Oncol. 2021 Mar 15;11:632181. doi: 10.3389/fonc.2021.632181 (PMC8005808; doi:10.3389/fonc.2021.632181)
Supplement: Supplementary file 1 [file DataSheet_1.docx]

Supplementary Material

# Supplementary Figures and Tables

## Supplementary Figure
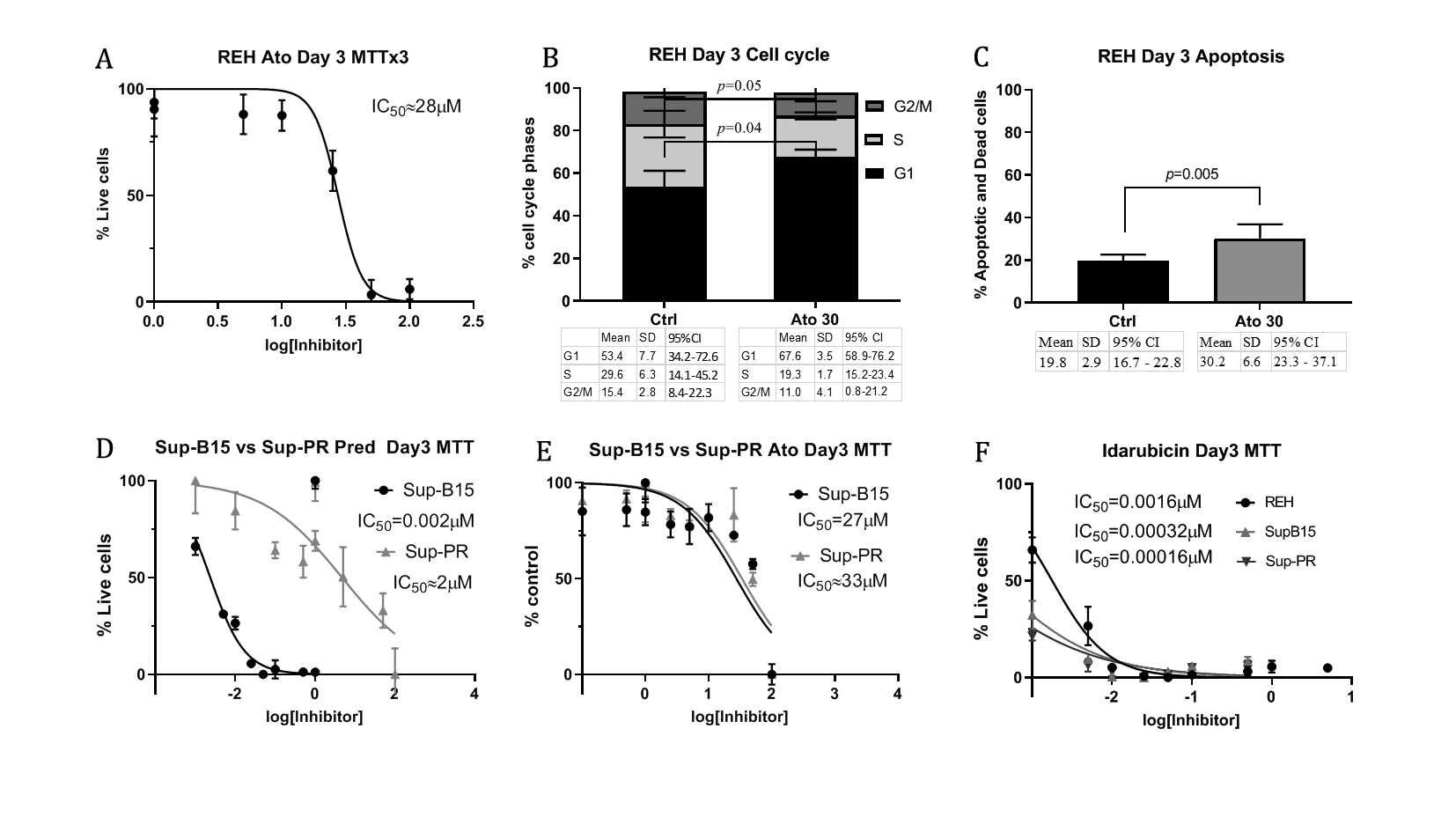


**Supplementary Figure 1**. A), D-F) MTT assays for determining IC_50_ values of Atovaquone in REH cells (A), Sup-B15 and Sup-PR cells (E), of IC_50_ values of Prednisolone in Sup-B15 and Prednisolone resistant Sup-PR cells (D), and of Idarubicin in REH, Sup-B15 and Sup-PR cells (F). Experiments were performed at least in biological duplicates and technical triplicates. Error bars represent SD of the mean. B) and C) Analysis of the effect of Atovaquone on cell cycle (B) and apoptosis (C) in REH cells. Data from biological triplicates of control and Ato-treated cells on day 3 were obtained from Guava® Muse® Cell Analyzer (Agilent) and cell cycle or AnnexinV/PI kit and plotted as shown. P-values were calculated with Student’s *t*-test; SD, standard deviation; 95%CI, 95% confidence interval.


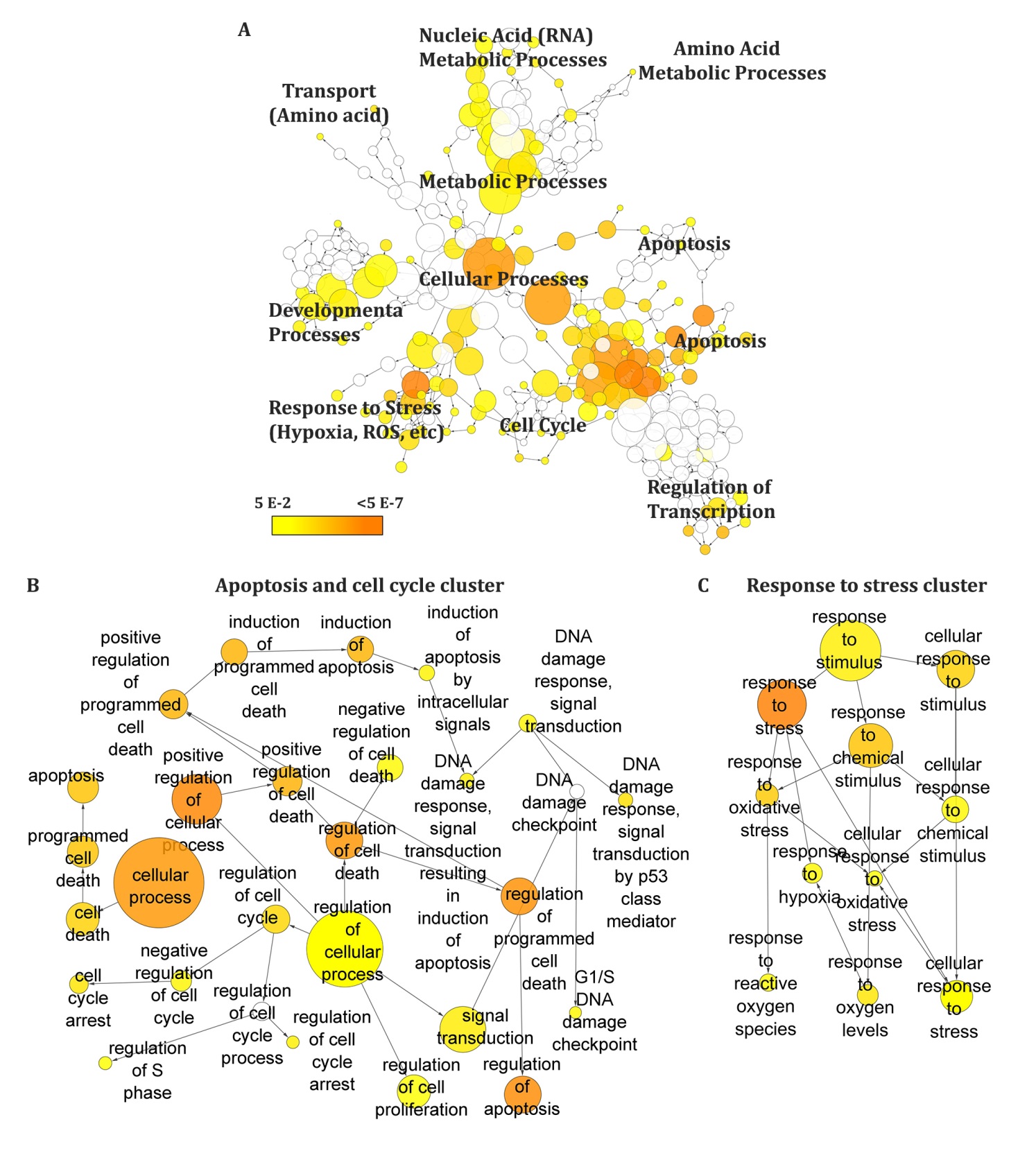


**Supplementary Figure 2**. GO analysis of RNA-seq data comparing control and Ato-treated REH cells taken on day 3 was performed with BinGO and Cytoscape. Gene list of significantly up- and down-regulated genes (FC >1.3, adjusted *p*-value<0.05) were used for the analysis. A) Overview of all clusters of enriched nodes. B) and C) Analysed clusters (removed statistically insignificant nodes) with nodes related to Apoptosis and cell cycle control (B) and Response to Stress (C). Colour represents degree of statistical significance of the respective node as calculated by BinGO.


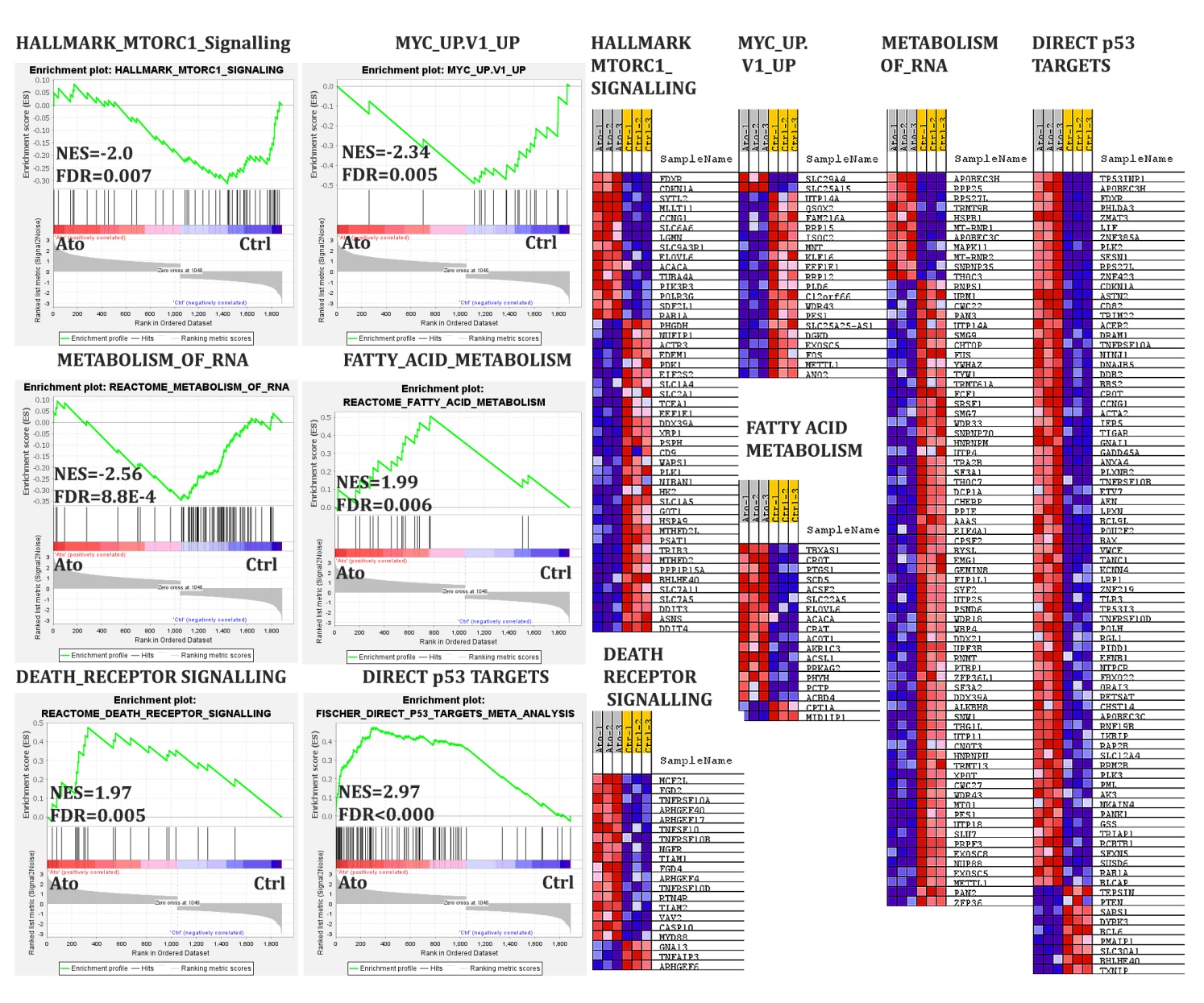


**Supplementary Figure 3.** GSEA data comparing Ato-treated to control cells showing significantly enriched gene sets and pathways as annotated. Heatmaps with up- (red) or down-regulated (blue) genes involved in the respective gene sets are shown. NES – normalised enrichment score; FDR – false discovery rate q-value.

## Supplementary Tables

|  | **REH Ctrl** | | | **REH Ato 30** | | | **Ctrl vs Ato** |
| --- | --- | --- | --- | --- | --- | --- | --- |
| **OCR (pmol/min/Norm. Unit)** | **Mean** | **SD** | **95% CI** | **Mean** | **SD** | **95% CI** | ***p*-value** |
| **Basal Respiration** | 407.6 | 48.8 | 458.8 - 356.4 | 219.4 | 53.9 | 276.0 - 162.8 | <0.0001 |
| **Maximal Respiration** | 813.8 | 93.1 | 911.5 - 716.1 | 220.2 | 116 | 342.1 - 98.3 | <0.0001 |
| **Spare Resp. Capacity** | 406.2 | 52.1 | 460.8 - 351.5 | 0.8 | 63.6 | 67.5 - -65.9 | <0.0001 |
| **ATP Production** | 343.0 | 32.3 | 376.8 - 309.1 | 137.6 | 79.6 | 221.1 - 54.1 | 0.0002 |

**Supplementary Table 1. Descriptive statistics of the effect of Atovaquone on REH cells as measured by Seahorse XFp Analyser and presented on Figure 1B**

SD-standard deviation, 95% CI – 95% confidence interval

**Supplementary Table 2. Top 50 upregulated and top 50 downregulated genes by Atovaquone**

|  | Upregulated by Ato | | |  | Downregulated by Ato | | |
| --- | --- | --- | --- | --- | --- | --- | --- |
|  | gene_name | log2FoldChange | padj |  | gene_name | log2FoldChange | padj |
| 1 | IFI44L | 2,91 | 2,45E-13 | 1 | CH25H | -4,65 | 1,65E-06 |
| 2 | NAPRT | 2,80 | 2,66E-02 | 2 | B3GALNT2 | -2,87 | 1,03E-18 |
| 3 | LIF | 2,70 | 3,28E-05 | 3 | JUN | -2,64 | 3,65E-07 |
| 4 | PHLDA3 | 2,52 | 2,65E-32 | 4 | PTEN | -2,10 | 1,84E-03 |
| 5 | HLA-DPB1 | 2,41 | 4,87E-04 | 5 | NOP9 | -2,05 | 6,88E-12 |
| 6 | APOBEC3H | 2,35 | 4,17E-12 | 6 | TXNIP | -2,05 | 4,60E-55 |
| 7 | TP53INP1 | 2,20 | 1,07E-38 | 7 | AC116025.2 | -1,92 | 1,28E-14 |
| 8 | EGF | 2,16 | 1,75E-06 | 8 | KLF2 | -1,87 | 2,34E-05 |
| 9 | MGAT3 | 2,12 | 1,24E-08 | 9 | PTPRK | -1,81 | 1,21E-02 |
| 10 | CCL25 | 2,09 | 1,83E-04 | 10 | SVILP1 | -1,80 | 4,38E-03 |
| 11 | CYBRD1 | 2,04 | 4,49E-05 | 11 | GDAP1L1 | -1,77 | 1,26E-06 |
| 12 | METTL7A | 1,97 | 6,33E-22 | 12 | ATOH8 | -1,77 | 1,96E-03 |
| 13 | SLC2A5 | 1,95 | 7,41E-05 | 13 | UNC5B | -1,75 | 1,23E-04 |
| 14 | GBP1P1 | 1,93 | 4,53E-07 | 14 | LPAR4 | -1,74 | 8,83E-08 |
| 15 | TNFSF9 | 1,91 | 1,13E-13 | 15 | TSC22D3 | -1,71 | 4,47E-29 |
| 16 | CD274 | 1,89 | 5,51E-04 | 16 | ARHGEF28 | -1,71 | 8,87E-03 |
| 17 | EHD3 | 1,87 | 9,39E-05 | 17 | HIST1H2BH | -1,68 | 1,31E-12 |
| 18 | CORO2A | 1,84 | 2,96E-11 | 18 | GBA3 | -1,68 | 4,80E-25 |
| 19 | ITGAL | 1,81 | 5,95E-03 | 19 | FOS | -1,68 | 3,96E-05 |
| 20 | A2M | 1,80 | 1,27E-02 | 20 | BFAR | -1,62 | 2,85E-07 |
| 21 | FDXR | 1,79 | 2,38E-28 | 21 | AL162726,3 | -1,61 | 1,07E-03 |
| 22 | PURPL | 1,78 | 7,04E-21 | 22 | GABRR2 | -1,59 | 2,52E-03 |
| 23 | INPP1 | 1,76 | 3,54E-03 | 23 | SERINC2 | -1,56 | 3,06E-07 |
| 24 | CDS1 | 1,76 | 6,50E-03 | 24 | PPP1R15A | -1,55 | 2,02E-05 |
| 25 | MYO1F | 1,73 | 1,23E-02 | 25 | INHBE | -1,55 | 6,52E-10 |
| 26 | GJA5 | 1,73 | 1,52E-04 | 26 | H1F0 | -1,54 | 7,84E-45 |
| 27 | NCF1C | 1,73 | 2,85E-04 | 27 | LINC01225 | -1,53 | 3,37E-05 |
| 28 | C1R | 1,71 | 9,22E-17 | 28 | TLE1P1 | -1,53 | 6,28E-06 |
| 29 | BRD2 | 1,70 | 4,93E-02 | 29 | ARPP21-AS1 | -1,52 | 3,57E-04 |
| 30 | AC090241,2 | 1,65 | 9,83E-03 | 30 | PINX1 | -1,51 | 4,58E-02 |
| 31 | EPHB3 | 1,64 | 1,06E-04 | 31 | IQCH | -1,51 | 1,42E-02 |
| 32 | MAGED4 | 1,63 | 3,49E-02 | 32 | CTGF | -1,49 | 1,08E-04 |
| 33 | PLK2 | 1,62 | 8,84E-13 | 33 | ANO2 | -1,47 | 1,38E-09 |
| 34 | FGD2 | 1,62 | 1,11E-03 | 34 | LINC02532 | -1,47 | 4,88E-04 |
| 35 | ELFN2 | 1,62 | 3,63E-05 | 35 | TRPC4 | -1,46 | 4,03E-10 |
| 36 | NCF1B | 1,59 | 7,00E-03 | 36 | BEST1 | -1,46 | 4,95E-02 |
| 37 | SRGN | 1,58 | 1,43E-05 | 37 | AC092384,1 | -1,44 | 4,44E-03 |
| 38 | EIF4E3 | 1,55 | 4,40E-06 | 38 | LRRC14B | -1,41 | 4,00E-22 |
| 39 | CHI3L2 | 1,53 | 8,00E-05 | 39 | TLR7 | -1,41 | 8,23E-04 |
| 40 | ZMAT3 | 1,53 | 1,90E-49 | 40 | COLGALT2 | -1,41 | 1,57E-02 |
| 41 | ZNF385A | 1,53 | 7,02E-13 | 41 | CDR2 | -1,41 | 1,21E-02 |
| 42 | DDO | 1,52 | 9,68E-03 | 42 | AL136084,2 | -1,41 | 5,43E-03 |
| 43 | SIK1 | 1,51 | 3,18E-02 | 43 | MRC1 | -1,41 | 8,53E-04 |
| 44 | FAM49A | 1,50 | 1,70E-26 | 44 | CEBPB | -1,41 | 3,60E-24 |
| 45 | ACTA2 | 1,50 | 1,22E-02 | 45 | APCDD1 | -1,40 | 7,65E-03 |
| 46 | CD70 | 1,50 | 5,91E-18 | 46 | AC138356,1 | -1,40 | 2,74E-03 |
| 47 | PLXDC1 | 1,49 | 3,40E-03 | 47 | ANPEP | -1,39 | 4,88E-02 |
| 48 | ZNF815P | 1,49 | 2,99E-03 | 48 | DDIT4 | -1,39 | 3,18E-11 |
| 49 | CD300LF | 1,47 | 1,12E-02 | 49 | ARRDC4 | -1,39 | 6,73E-06 |
| 50 | ERVH48-1 | 1,47 | 3,74E-04 | 50 | BX276092,7 | -1,38 | 2,38E-02 |
|  |  |  |  | 59 | VEGFA | -1,32 | 2,66E-19 |

**Supplementary Table 3. Descriptive statistics of the effect of Atovaquone on Sup-B15 and Sup-PR cells as measured by Seahorse XFp Analyser and presented on Figure 2B**

|  | **Sup-B15** | | | **Sup-B15 Ato 30** | | | **Ctrl vs Ato** |
| --- | --- | --- | --- | --- | --- | --- | --- |
| **OCR (pmol/min/Norm.Unit)** | **Mean** | **SD** | **95% CI** | **Mean** | **SD** | **95% CI** | ***p*-value** |
| **Basal Respiration** | 698.3 | 42.5 | 592.8 - 803.7 | 396.4 | 46.6 | 280.7 - 512.1 | 0.0012 |
| **Maximal Respiration** | 401.7 | 39.3 | 304.2 - 499.2 | 293.7 | 52.9 | 162.3 - 425.1 | 0.046 |
| **ATP Production** | 603.2 | 16.8 | 561.6 - 644.8 | 297.7 | 70.4 | 122.8 - 472.7 | 0.0019 |
|  | **Sup-PR** | | | **Sup-PR Ato 30** | | | **Ctrl vs Ato** |
| **OCR (pmol/min/Norm.Unit)** | **Mean** | **SD** | **95% CI** | **Mean** | **SD** | **95% CI** | ***p*-value** |
| **Basal Respiration** | 336.6 | 46.7 | 220.5 - 452.6 | 236.2 | 15.0 | 199.0 - 273.4 | 0.024 |
| **Maximal Respiration** | 261.4 | 22.7 | 205.0 - 317.8 | 122.4 | 53.7 | -11.1 - 255.8 | 0.015 |
| **ATP Production** | 279.6 | 44.7 | 168.5 - 390.7 | 156.7 | 4.6 | 145.4 - 168.0 | 0.009 |

SD-standard deviation, 95% CI – 95% confidence interval

**Supplementary Table 4 Descriptive statistics of cell growth effect of Ato and Idarubicin on REH cells (x10^5^ number of cells) as shown on Figure 3B**

|  | **Mean** | **SD** | **95% CI** |
| --- | --- | --- | --- |
| **Ctrl** | 1.96 | 0.40 | 1.66 - 2.27 |
| **Ato** | 1.45 | 0.38 | 1.16 - 1.74 |
| **Ida** | 1.42 | 0.30 | 1.17 - 1.68 |
| **Ato+Ida** | 0.83 | 0.19 | 0.68 - 0.97 |

SD-standard deviation, 95% CI – 95% confidence interval

**Supplementary Table 5. Patient sample information**

| **Patient sample #** | **Type of ALL** | **Age** | **Gender** | **Genetic profile** | **% blast count in BM at diagnosis** | **Risk stratification** |
| --- | --- | --- | --- | --- | --- | --- |
| 1 | B-ALL | 11 | Male | +ve for t(12;21)  -ve for t(9;22), t(4;11), t(1;19), t(8;14) | 85% | High risk* |
| 2 | B-ALL | 7 | Female | +ve for t(12;21)  -ve for t(9;22), t(4;11), t(1;19), t(8;14) | 90.6% | Standard risk |
| 3 | B-ALL | 3 | Male | +ve for t(12;21)  -ve for t(9;22), t(4;11), t(1;19), t(8;14) | 95% | Standard risk |
| 4 | B-ALL | 3 | Female | +ve for t(12;21)  -ve for t(9;22), t(4;11), t(1;19), t(8;14) | 91.5% | Standard risk |

*Initially standard risk, but with poor response during induction

t(12;21) – TEL-AML1 fusion, t(9;22) – BCR-ABL fusion, t(4;11) – MLL-AF4 fusion, t(1;19)-TCF3-PBX1 fusion, t(8;14) –IGH-MYC fusion

**Supplementary Table 6. Statistics for the effect of Atovaquone on patient samples as shown on Figure 4C**

|  | **Control** | | | **Ato** | | | **Ctrl vs Ato** |
| --- | --- | --- | --- | --- | --- | --- | --- |
| **OCR (pmol/min/Norm.Unit)** | **Mean** | **SD** | **95% CI** | **Mean** | **SD** | **95% CI** | ***p*-value** |
| **Basal Respiration** | 3.9 | 1.1 | 2.14 - 5.66 | 0.2 | 1.2 | -1.69 - 2.06 | 0.04 |
| **Maximal Respiration** | 11.4 | 4.7 | 3.85 - 18.9 | 0.4 | 2.0 | -2.76 - 3.50 | 0.042 |
| **ATP Production** | 4.0 | 1.1 | 2.27 - 5.67 | -0.3 | 2.2 | -3.85 - 3.18 | 0.028 |

SD-standard deviation, 95% CI – 95% confidence interval
